# Saccade execution increases the preview effect with faces: An EEG and eye-tracking coregistration study

**DOI:** 10.3758/s13414-023-02802-5

**Published:** 2023-11-02

**Authors:** Christoph Huber-Huber, David Melcher

**Affiliations:** 1 Center for Mind/Brain Sciences (CIMeC), University of Trento, Corso Bettini 31, 38068 Rovereto, Italy; 2 Center for Brain & Health, New York University Abu Dhabi, Abu Dhabi, United Arab Emirates; 3 Psychology Program, Division of Science, New York University Abu Dhabi, Abu Dhabi, United Arab Emirates

**Keywords:** Perception and action, Eye movements, Methods: VEP, EEG, fixation-related potentials

## Abstract

Under naturalistic viewing conditions, humans conduct about three to four saccadic eye movements per second. These dynamics imply that in real life, humans rarely see something completely new; there is usually a preview of the upcoming foveal input from extrafoveal regions of the visual field. In line with results from the field of reading research, we have shown with EEG and eye-tracking coregistration that an extrafoveal preview also affects postsaccadic visual object processing and facilitates discrimination. Here, we ask whether this preview effect in the fixation-locked N170, and in manual responses to the postsaccadic target face (tilt discrimination), requires saccade execution. Participants performed a gaze-contingent experiment in which extrafoveal face images could change their orientation during a saccade directed to them. In a control block, participants maintained stable gaze throughout the experiment and the extrafoveal face reappeared foveally after a simulated saccade latency. Compared with this no-saccade condition, the neural and the behavioral preview effects were much larger in the saccade condition. We also found shorter first fixation durations after an invalid preview, which is in contrast to reading studies. We interpret the increased preview effect under saccade execution as the result of the additional sensorimotor processes that come with gaze behavior compared with visual perception under stable fixation. In addition, our findings call into question whether EEG studies with fixed gaze capture key properties and dynamics of active, natural vision.

## Introduction

One of the most powerful capacities of the human brain is the ability to detect regularities and contextual clues in the environment and to use that learning to predict future events. Many recent theories recognize a central role for prediction in brain function (Clark, 2013; de Lange et al., 2018; Friston, 2005). The theoretical focus on predictive and active aspects of brain function and cognition is, however, often at odds with the way in which brain and cognition are studied empirically. Historically, neuroscientific research in visual perception and word reading has started with a more passive model, using experimental paradigms in which words, faces or other visual stimuli suddenly appear at the center of gaze. In natural viewing, however, stimuli very rarely just materialize out of nowhere. Instead, the onset of a stimulus at the fovea is typically the result of having made a saccadic eye movement toward a target of interest. This discrepancy raises the question whether the classic experimental setup with fixed gaze is actually a good model of visual perception. Here, we present a study which suggests that the classic model fails to capture important aspects of naturalistic vision. We show that active gaze behavior affects visual processing at an early postsaccadic stage, in a way that has behavioral consequences.

Theories of active vision highlight that our sensory systems can take advantage of the ability of the brain to use information from the oculomotor system to predict “what” will appear on the retina, “where” the stimulus will appear with respect to the fovea, and “when” this abrupt onset will occur in time (for review Melcher & Colby, 2008; see also Auksztulewicz et al., 2018; Melcher & Morrone, 2003; Schroeder et al., 2010). Given that people make thousands of saccades every day, it would perhaps be surprising if the visual system did not take advantage of these trans-saccadic regularities. Indeed, in the past 20 years, there have been a growing number of studies showing that presaccadic visual information influences postsaccadic processing of visual features such as shape (Demeyer et al., 2010; Fracasso et al., 2010; Gordon et al., 2008; Harrison & Bex, 2014; Melcher, 2005, 2007; Prime et al., 2011; Van Eccelpoel et al., 2008; Wolfe & Whitney, 2015; Zimmermann et al., 2013; Zirnsak et al., 2011) and color (Wijdenes et al., 2015; Wittenberg et al., 2008) see also (Herwig, 2015; Herwig & Schneider, 2014; Paeye et al., 2018).

How might these particular aspects of trans-saccadic processing influence every day visual perception? One example is reading, where we use information from the extrafoveal visual field (i.e., an upcoming word, before making a saccade to that word to process it foveally). The interactions of extrafoveal, usually parafoveal, and subsequent foveal processing in reading have traditionally been studied with the preview paradigm where an upcoming word is manipulated in a gaze-contingent way during the saccadic eye movement that is made to that word in order to create a condition in which the presaccadic extrafoveal input is different from the following postsaccadic foveal input. Such an invalid preview condition is then usually compared with a valid preview condition, in which there is no change across the saccade (for reviews, see Himmelstoss et al., 2020; Huber-Huber et al., 2021a; Rayner, 1998; Schotter, 2018; Schotter et al., 2012). It is typically reported that postsaccadic word processing is more efficient in valid compared with invalid preview conditions, which led to the terminology of a *preview benefit* effect (however see Kliegl et al., 2013; Marx et al., 2016, for a discussion whether the term *benefit* is appropriate). Studies using the preview paradigm have revealed that parafoveal word recognition starts at multiple levels already, before we directly foveate the word, although the relative composition and extent to which semantic, linguistic, and visual word-related features of a preview can be extracted is still a matter of debate (Hohenstein & Kliegl, 2014; Pan et al., 2020; Schotter & Fennell, 2019). This early onset of word recognition before direct fixation is in line with the idea that imminent foveal input is constantly being predicted as part of the interplay between visual-sensory and oculomotor systems.

In recent years, it has become increasingly common to coregister brain activity (EEG, MEG, fMRI) with eye tracking in order to obtain more detailed insights into visual processing in more ecologically valid experimental setups that allow for active gaze behavior (Auerbach-Asch et al., 2020; Degno et al., 2019; Dimigen et al., 2012; Nikolaev et al., 2016; Schuster et al., 2020, 2021; Sereno & Rayner, 2003; see Hutzler et al., 2007, for pioneering work). Coregistering EEG with eye tracking has provided detailed insights into the time course of trans-saccadic processing and, in particular, into when exactly preview information modulates postsaccadic processing. For instance, there is evidence that the influence of preview information during reading is not continuously present throughout a fixation but instead specifically affects postsaccadic processing primarily around 200–300 ms after the onset of a new fixation (Dimigen et al., 2012). This time frame is similar, but perhaps slightly earlier, for face perception, where we found that the face-related preview information affects the face-sensitive N170 component elicited by the fixation on a target face (Buonocore et al., 2020; Huber-Huber et al., 2019). The classic N170 component is generally considered as a hallmark of visual face processing. It has a bilateral posterior distribution, is strongest over electrode locations such as P7/8 or PO7/8, and peaks usually around 170 ms after stimulus onset. It is modulated by various aspects of face processing and is commonly thought to indicate the detection of a face, or face-like features, in contrast to other visual input. It is also usually more pronounced and delayed for inverted compared with upright faces (Rossion & Jacques, 2011). The effect that we have repeatedly found in the fixation-locked N170 consisted primarily in a reduced amplitude in valid compared with invalid preview conditions in which faces were scrambled or presented upside-down until the participant fixates directly on the face (Buonocore et al., 2020; Huber-Huber et al., 2019). This preview effect is consistent with the conjecture of other researchers that the N170 could reflect predictive visual perception across eye movements (Johnston et al., 2017). Except from one study with gratings (Ehinger et al., 2015), neural preview effects have so far been mainly investigated in the field of face perception (Buonocore et al., 2020; de Lissa et al., 2019; Dimigen et al., 2012; Edwards et al., 2018; Huber-Huber et al., 2019) and for reading research (Li et al., 2015; Niefind & Dimigen, 2016).

How the presaccadic preview affects postsaccadic processing and behavior is, however, not yet fully understood and this is complicated by the fact that visual perception varies substantially across the visual field due to the inhomogeneous distribution of rods and cones. Visual input from the extrafoveal visual field where preview information is available is strikingly different from the visual input created by the same stimulus in the foveal field of view (e.g. Huber-Huber et al., 2021a; Liu et al., 2023). Here, the central idea of active vision is that saccadic eye movements allow the visual system to learn how extrafoveal visual input maps onto postsaccadic foveal input and that, therefore, saccadic eye movements are considered to be crucial for predictive processing in active vision (cf. Herwig & Schneider, 2014; Valsecchi & Gegenfurtner, 2016).

The extrafoveal preview that comes with active gaze behavior affects foveal visual processing; however, critics of the active perception perspective have raised the question whether active gaze is necessary for preview effects. The mere finding of a preview effect with saccades does not mean that this effect is caused specifically by the sensorimotor contingencies of active gaze behavior. It is also possible that the preview effect reflects some repetition facilitation that simply results from the fact that the visual system is confronted with the same input—albeit first extrafoveally and then foveally—twice in quick succession. The saccadic eye movement that maps the extrafoveal input to subsequent foveal input might be sufficient, but not necessary, for a preview effect.

Following the argument that a trans-saccadic facilitation effect might in theory not be specific to active gaze behavior but could simply result from repetition, many trans-saccadic perception studies have included corresponding control conditions. Some evidence for a role of saccade planning in the preview effect for words, for instance, comes from a study comparing natural reading to rapid serial presentation (RSVP) of words on the retina (Kornrumpf et al., 2016). In the RSVP paradigm, the word itself was moved from the periphery to the fovea while eyes remain fixated. Thus, the foveal input was matched across conditions, with the only difference that in the natural reading condition the onset of the new word was caused by a saccade. In the natural reading condition, the authors replicated the preview effect, with a greatly reduced evoked response after valid compared with after invalid previews. In the RSVP condition, however, the preview effect was substantially reduced. This suggests that, at least in the case of reading, the preview effect is directly related to the sensorimotor processes involved in active gaze behavior.

An early study on preview during reading featured similar control conditions in which the visual input during active gaze behavior was—as well as possible—matched with a no-saccade control condition in which the eyes remained stable (Rayner et al., 1978). Interestingly, however, the researchers arrived at the opposite conclusion. They found very similar preview effects in a simulated saccade condition, which made them conclude that active gaze behavior is not required for preview effects. According to them, a saccade does not do much else than bringing the extrafoveal visual information into the center of the visual field, which does not involve any anticipatory processing. Importantly, they measured the influence of the preview on postsaccadic processing with a behavioral task. Participants had to name the words that they were looking at. Measuring a preview effect with this task is very different from measuring a preview effect in the early fixation-locked potential of the EEG. A word-naming task entails many additional processes. Thus, one explanation for this discrepant pattern of results seems to be that visual word recognition during reading is supported by anticipatory mechanisms that are tightly linked to the oculomotor loop but these anticipatory mechanisms have less of an effect at the later stage of language production, in the sense of reading text out loud.

Other studies on trans-saccadic perception included similar control conditions and arrived at mixed conclusions regarding the relevance of active gaze behavior (Bompas & O’Regan, 2006; Ganmor et al., 2015; Grzeczkowski et al., 2020; Paeye et al., 2017). For instance, Herwig and Schneider (2014) investigated, in their Experiment 2, whether the trans-saccadic association effect of object shape and spatial frequency also occurred if participants did not move their eyes. Participants judged the spatial frequency of certain geometrically shaped objects which could systematically change during the cued eye-movement that participants made towards the objects. Indeed, in contrast to the saccade condition, in a condition with fixed gaze spatial frequency judgements were not significantly biased towards the consistent trans-saccadic changes, which suggests that active gaze behavior is necessary to incorporate predictable trans-saccadic changes into visual perception. On the other hand, Paeye et al. (2018) arrived at the opposite conclusion for a very similar effect. In their study, shape perception judgements adapted to predictive trans-saccadic changes in an active saccade condition and in a no-saccade control condition the effect was also present, albeit significantly smaller. A very similar pattern of results is reported by Valsecchi and Gegenfurtner (2016) in a size perception task. Participants showed a comparable, but less stable, bias to predictable trans-saccadic changes in stimulus size when stimuli moved from the visual periphery to the center of gaze compared with when participants executed saccades towards the stimulus. These examples from other trans-saccadic perception studies already demonstrate that many effects that appear to be specific to active gaze behavior can also be observed in the absence of eye movements, albeit often in smaller size.

The present study was conducted to determine whether the trans-saccadic preview effect with faces (Huber-Huber et al., 2019) depends on actually making a saccade. We designed an experiment with a preview manipulation (valid, invalid) similar to our previous experiments, with the change that there were now two different viewing conditions, one in which participants made a cued saccade to an extrafoveal face and one in which we matched the visual input to the saccade condition but the participants’ eyes remained at the central screen location throughout each trial. In this no-saccade control condition we simulated the participants’ saccade latencies and durations and presented the extrafoveal preview face after randomly determined time periods again at the foveal screen center while participants maintained stable fixation.

Previous studies that investigated whether trans-saccadic perceptual effects are specific to actual saccade execution followed two different lines of argument and consequently employed two different types of designs for the no-saccade control condition. First, one could argue that the no-saccade condition should match the saccade condition in all of its spatial and temporal aspects to recreate exactly the same statistical regularities and stimulation parameters. Following this reason, one would design a no-saccade condition in which the timing of the onset of the foveal target stimulus is matched to the saccade latencies and durations of the saccade condition, as has been done by some researchers (Cox et al., 2005; Herwig & Schneider, 2014). Second, one could argue that the first strategy means that the simulated foveal target onset is temporally not predictable but the onset of fixation on the target in the saccade condition is predictable and in order to equate the predictability between the both viewing conditions, the no-saccade control condition should have a temporally predictable foveal target onset. Following this line of argument, one would design the no-saccade condition with a constant simulated saccade latency, perhaps adjusted to individual participants’ average or median saccade latencies, as has been done by other researchers (Kornrumpf et al., 2016; Paeye et al., 2018). We followed the first approach and randomly drew a saccade latency value for each trial based on each participants’ saccade data from the practice block.

We manipulated preview validity by turning the preview face upside down on invalid trials. That means, in the invalid preview condition the preview face was inverted and in the valid preview condition the preview face was upright. The target face was always upright. In our previous study with upright and inverted faces, this manipulation was slightly different: Not only could the preview face could be upright or inverted, but also the target face could change orientation. To reduce the complexity and increase the power of the current experimental design, which featured an additional no-saccade control condition, we decided to omit inverted targets and always present the target face upright. In principle, this change might make it more difficult to find a preview effect, in that participants can actually predict that the target will always be upright and could strategically ignoring the preview stimulus altogether which might abolish any postsaccadic preview effect. However, this design decision renders our experimental design more similar to studies on the preview effect in the area of reading research which usually only have one type of target stimulus and different preview stimuli (e.g., a degraded versus an intact preview word; Dimigen et al., 2011). Considering that reading studies consistently report a preview effect (Dimigen et al., 2011; Schotter et al., 2012; Vasilev & Angele, 2017), we reasoned that we would still find a preview effect with our design with exclusively upright targets. Moreover, finding a preview effect with only upright targets would be a sign for a more automatic preview process and because we want to know whether there is a preview effect also in a passive no-saccade condition, which is supposedly more conducive to automatic processes compared with an active saccade condition, employing an experimental design that aims at a more automatic preview effect might even present overall a more conservative test.

We hypothesized that the preview effect would be modulated by whether the participants actually made a saccade to the extrafoveal target. In particular, we expected that the difference in the fixation-related N170 between valid and invalid would be larger in the saccade condition compared with the no-saccade control condition, similar to what Kornrumpf et al. (2016) found for the preview effect in reading. We expected the same pattern of results for the behavioral preview effect in the tilt discrimination task on the target face. To decide whether saccades are not only sufficient, but also necessary, for a strong preview effect, it is not only important to see whether the preview effect is larger in the saccade than in the no-saccade condition, but also whether there is any significant preview effect in the no-saccade condition. If there was still a preview effect in the no-saccade condition, although perhaps a very small one, saccades would not be strictly necessary for a preview effect. If there was no preview effect in the no-saccade condition, we could indeed conclude that only active gaze behavior provides the crucial information about upcoming visual input which eventually leads to preview effects. However, even if there was a preview effect also in the no-saccade condition, but a larger one in the saccade condition, active gaze behavior would still matter and the difference in the preview effect between both viewing conditions would be a measure of the extent of anticipatory processing that is implied by saccade execution. In contrast, if the preview effect was exactly the same in saccade and no-saccade conditions, or any larger without saccades, then this would suggest that the preview effect results exclusively from a rather passive function of the visual system. In that case, the link between active gaze behavior and predictive processing within the visual system would have to be considered less tight than previously suggested.

We measured the behavioral preview effect in a postsaccadic perceptual task and the neural preview effect in the early fixation-related and event-related EEG response. We already know that these two effects do not necessarily reflect the same mechanisms, because they are differentially susceptible to environmental statistical regularities (Huber-Huber et al., 2019). This consideration is important to keep in mind when investigating neural and behavioral preview effects in the same experiment. In addition, here, we also measured a preview effect in gaze behavior. The first fixation duration on a target stimulus has consistently been reported to be reduced after valid preview compared with invalid preview in reading studies (Vasilev & Angele, 2017). To see whether this translates to vision more generally, we analyzed the first fixation duration on the target face in our dataset. The second reason to further analyze gaze behavior was to rule out any confounds with respect to neural preview effects. For this purpose, we additionally examined saccade latencies and amplitudes because in particular saccade amplitudes are known to strongly affect the fixation-related potential (Dimigen et al., 2011; Kaunitz et al., 2014; Ries et al., 2018).

## Methods

### Participants

Concurrent EEG and eye-tracking data was recorded from 26 participants with normal or corrected-to-normal vision. Fourteen of them identified as female, 12 as male. Three were left-handed and 11 were left-eye dominant. The mean age was 25 years, ranging from 19 to 42 years. Written informed consent was obtained prior to the experiment in line with Declaration of Helsinki and the participants received monetary reimbursement for their efforts. The study was approved by the ethics committee of the University of Trento.

### Apparatus and stimuli

The experiment was programmed with the Psychophysics Toolbox (Brainard, 1997; Pelli, 1997) in MATLAB (Version 2014b, The MathWorks Inc.). We used the same 16 face stimuli, and their phase-scrambled counterparts, as in a previous experiment (Huber-Huber & Melcher, 2021). As described previously, stimuli were originally selected from the Nottingham face database (http://pics.stir.ac.uk/zips/nottingham.zip) and adjusted to fit the current demands. A circular mask with a diameter of 2.88° of visual angle was placed on the faces in a way that the eyes, eyebrows, nose, and mouth were about equally well represented in each image. Average luminance histograms were equated across images with the SHINE toolbox (Willenbockel et al., 2010). The images were presented at 8° of visual angle eccentricity to the left of a central 0.5° by 0.5° fixation cross on a ViewPixx monitor specifically designed for vision research (ViewPixx/EEG, VPX-VPX-2006A, by VPixx Technologies Inc., Saint-Bruno, Ontario, Canada), running at 120 Hz screen refresh rate. Upright faces served as foveal targets and upright faces as well as their inverted counterparts served as extrafoveal previews to create valid (upright face) and invalid (inverted face) preview conditions.

Presenting faces only in the left visual field poses a very different situation for covert and pre-saccadic attentional processing compared to presenting faces both in the left and in the right visual field. Our previous work has, however, shown that the preview effect in the N170 time period is the same for both kinds of designs (compare Experiment 1 and 2 in Huber-Huber et al., 2019). We, therefore, presented faces only on one side of the visual field to reduce the complexity of the experimental design. We presented the faces in the left visual hemifield, because our previous work with faces presented on both sides of the visual field also showed a slightly noisier behavioral effect for faces on the right side than on the left side which hint at a purported right-lateralization of face processing (e.g. Sergent, Ohta, & Macdonald, 1992). This conjecture is, however, very speculative and deserves further investigation. When comparing the preview effect with faces to preview effects in reading research, it is important to keep in mind that, although reading preview studies present only one preview stimulus like in our design, this preview stimulus is usually in the right visual field (for a review see Schotter et al., 2012).

### Procedure and design

**Fig. 1:**
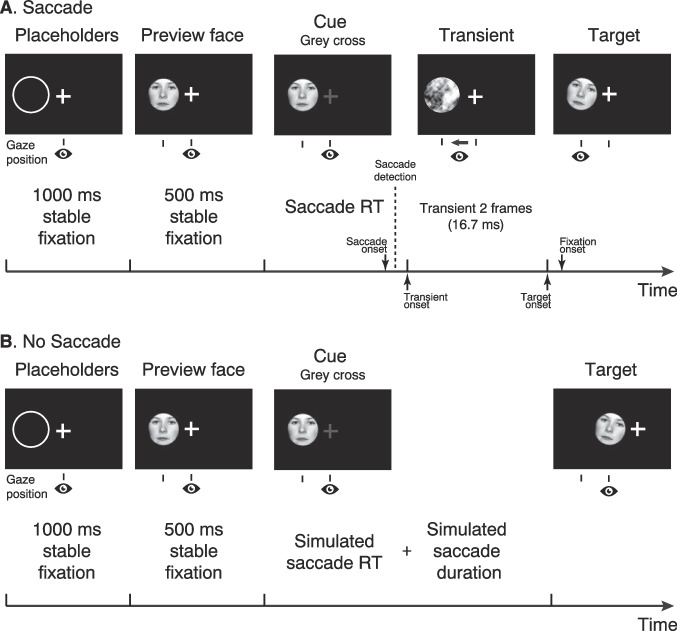
Experimental procedure in the saccade (**A**) and no-saccade (**B**) blocks. In both blocks, a trial started with a placeholder, followed by the preview face and the saccade cue. Here, the preview face is shown upright. In both blocks, the preview face was inverted (upside-down) in half of the trials to create invalid preview conditions (not illustrated). The visual transient was only presented in the saccade condition, whereas in the no-saccade condition, the preview face remained on screen until the screen changed to the target display. The eye icon below the image sequence illustrates the participant’s gaze position which changed from the center to the left of the screen in the saccade condition and remained at the center of the screen in the no-saccade condition. The time line at the bottom of each panel specifies the timing of events within a trial

The saccade and no-saccade conditions are illustrated in Fig. 1. For both the saccade and the no-saccade conditions, each trial started with a central fixation cross that had on its left a placeholder ring, width 1 pixel, of 2.88° diameter centered at the 8° eccentric location of the upcoming face image. As soon as the EyeLink eye tracker recorded fixation update events within 2° of the central fixation cross consecutively for 1 s, the extrafoveal preview face appeared on screen. In each trial, the preview face image was the same as the subsequent target, if the trial was valid, and it was the same face but inverted for the invalid preview condition. Another 500 ms of stable gaze made the fixation cross turn grey. This change in the fixation cross was the cue for the participants to make a saccade to the extrafoveal preview face in the saccade block of the experiment. In the no-saccade block, the cue turned grey, too, but the participants had been advised to keep their gaze at the fixation cross.

After the fixation cross turned grey in the saccade block, the saccade onset was detected online by a heuristic. If the eye-tracker recorded two subsequent gaze samples more than 0.18° apart, this counted as saccade and the phase-scrambled version of the target face was presented as an intrasaccadic transient, exactly as in our previous studies (Huber-Huber et al., 2019; Huber-Huber & Melcher, 2021). The purpose of the transient was to ensure that there was a similar change on the screen during the saccade in both valid and invalid trials. The transient was presented for two frames (i.e., 16.7 ms) and was followed by the target face. This procedure ensured that the target was presented before the participant’s eyes landed on the target location in most of the trials. Trials in which the target was presented more than 50 ms after the participants’ fixation onset were discarded in the analysis.

In the no-saccade block, there was no saccade detection. Instead, the participants had been instructed to maintain central fixation which was controlled online by the eye tracker with the same criterion as for the initial fixation (see above). If the participants deviated from the fixation cross, the trial restarted. In order to proceed, stable gaze was required for a time period of simulated saccade latency plus saccade duration. In each no-saccade trial, this time period was randomly drawn from the combination of an ex-Gaussian (latencies) and a Gaussian (durations) distribution that had been fitted to the saccade latencies and saccade durations of the initial practice block separately for each participant (for ex-Gaussian fitting, see Zandbelt, 2014). Summing up a randomly drawn saccade latency and a randomly drawn duration value yielded a simulated time point for the fixation onset in each trial (i.e., the time point at which to present the target stimulus). Note that, in contrast to a real fixation onset, the timing of target display presentation was constraint and discretized by the screen refresh rate (120 Hz). If the gaze was stable for the stimulated saccade latency time period, the extrafoveal preview image was removed and the target image appeared at the center of the screen (i.e., in the participants’ foveal field of view). At the same time, the fixation cross was shifted by the same distance to the right side to maintain the layout of the display.

In both the saccade and no-saccade blocks, the target face remained on screen for 800 ms and had a small tilt, randomly to the left or right, which had to be reported by the participants upon fixating the target by a manual response button on the computer keyboard. In order to obtain a high level of correct trials for all participants despite individual variation, the amount of tilt (i.e., the absolute deviation from 0° vertical) was adjusted by a staircase procedure (QUEST; Watson & Pelli, 1983). The log10 of the tilt angle was taken as the variable in the staircase algorithm in order to account for the fact that smaller deviations are more difficult to distinguish. The initial tilt angle was set to 1.8°, minimum to 0.5°, maximum to 10°. The guessed proportion correct at start, the so-called threshold, was .90, with step size 0.01. The standard deviation of the underlying Gamma distribution was set to 3 with beta 1.2, based on pilot data of one participant (not included in the analysis). Delta was 0.01 (fixed, default value), gamma 0.5 (necessarily, because of two-alternative forced choice). The staircase started with the first practice block and remained active until the end of the experiment. Note that, as in our previous studies (Huber-Huber et al., 2019; Huber-Huber & Melcher, 2021), the tilt was only present for the target face and absent from the preview face which rendered the preview stimulus actually task-irrelevant.

Saccade and no-saccade conditions were blocked and each part extended across half of the experiment, with the order counterbalanced across participants. Within each saccade/no-saccade part of the experiment, valid and invalid conditions were randomly intermixed. Participants performed 320 trials in each saccade/no-saccade part of the experiment. At the beginning of each part, participants were instructed about the upcoming condition. Each part was further divided in small blocks of 32 trials, between which participants could take a break for as long as they wanted. In total, the experiment consisted of 640 trials, 160 per cell of the preview validity (valid, invalid) by viewing condition (saccade, no-saccade) design. Within each cell, each of the 16 face images occurred exactly 10 times.

At the start of the experiment, the eye-tracker was calibrated with a 9-point grid. Throughout the experiment, the experimenter monitored the recorded gaze position online and was informed on a separate screen if the first target fixation was too far away from face center (more than 2°) or if the gaze did not remain on the target face (the same 2° threshold) until the response button was pressed. If the experimenter had the impression that the eye-tracker calibration was not correct anymore, the experiment was put on hold and the eye tracker was recalibrated.

After the initial calibration, each participant ran a practice block of 32 trials in each the saccade and the no-saccade conditions, with the same saccade/no-saccade order as in the following proper experiment. If participants had practiced the no-saccade condition at first, the simulated fixation onsets were based on the group-average saccadic response times from a previous experiment (Huber-Huber & Melcher, 2021) and for the following no-saccade trials of the proper experiment the simulations were based on the saccade practice block. During the practice trials, the participants received feedback to their manual responses. If a response was incorrect, the placeholder circle turned red for 270 ms at the end of a trial. There was no such feedback during the proper experiment.

### EEG and eye-tracking data recording and analysis

The electroencephalogram (EEG) was recorded with a 64-channel DC system (Brain Products GmbH, software: BrainVision Recorder Version 1.21) at 1000 Hz in an electromagnetically shielded booth. Electrode locations followed the 10–10 system, the ground electrode was placed at Cz and the online reference at the right mastoid.

Eye movements were recorded at the same sampling rate of 1000 Hz with a video-based EyeLink 1000 eye-tracker in desktop-mount mode (SR Research, Ontario, Canada) concurrently with the EEG. Saccade and fixation events were parsed from the continuous gaze position data with default settings (velocity threshold 35°/s, acceleration threshold 9500°/s2). To reduce the noise in the online signal, the online heuristic filter was set to level 2, which delays the online gaze data by a few milliseconds, but this tiny drawback was made up for by the higher online data quality and consequently more reliable online saccade detection.

Parallel port triggers were sent to both EEG and eye-tracking acquisition systems simultaneously by means of a splitter cable. To offline synchronize both data streams, we used the EYE-EEG add-on (Dimigen et al., 2011) to the EEGLAB toolbox (Version 14.1.1; Delorme & Makeig, 2004). All data processing was done in MATLAB (Version R2019b, The MathWorks Inc.).

After synchronizing EEG and eye-tracking data, the signals were down-sampled to 250 Hz, low-pass filtered (Hamming windowed sinc FIR filter, edge of the passband 40 Hz, transition band width 10 Hz, −6dB cutoff frequency 45 Hz), and visually inspected for bad channels, which were interpolated and finally rereferenced to the average reference because this reference is optimal for the face-related N170 component (Hinojosa et al., 2015). The continuous data were epoched into trials from −200 to 600 ms with respect to the first fixation onset on the target (saccade condition) or target onset (no-saccade condition). Baseline correction was performed based on the 200 ms prefixation/target period. In previous experiments, we took the 200 ms interval before preview face onset as baseline period (Huber-Huber et al., 2019). However, in the current design with only upright targets, we chose a baseline that is more in line with preview studies from the field of reading research (e.g. Kornrumpf et al., 2016). The rationale is that we only have upright targets, and if we took the baseline period from before the preview onset, the response to the (fixation onset on the) target would be confounded with the response to the preview itself. Taking the prefixation/target period as baseline instead corrects for any differences in signal offset due to the different preview conditions and the following postfixation/target EEG response becomes indicative of the change from preview to target face.

The epoched EEG data were visually inspected for major artifacts, and bad epochs were removed. In addition, we only included epochs in the analysis in which the participants followed the gaze procedure—that is, they initially maintained stable fixation within 2° of the screen center, made no saccades before cue onset in the saccade condition, and no saccades before target onset in the no-saccade condition. If the target had not been presented before fixation onset due to a delay in saccade detection, the time difference between fixation onset and target onset had to be less than 50 ms. In addition, saccadic response times to the gaze cue in the saccade condition and manual response times to the target tilt in both saccade and no-saccade conditions had to be within three median absolute deviations, separately for each participant. Trials with errors in the tilt discrimination task were excluded from all analyses, except for behavioral error rates. The set of trials for the EEG analysis was exactly the same as the set of trials for the manual response time and the gaze data analyses.

In order to remove as much eye-movement-related activity from the EEG signal as possible and thus equalize saccade and no-saccade conditions, we applied independent component analysis (ICA; Dimigen, 2020; Makeig et al., 1996). Note, however, that the effect of interest (i.e., the preview effect), consisted of a difference between two conditions, valid and invalid preview, which both had the same contribution from the oculomotor system because the saccade/no-saccade task was the same for both conditions within each saccade or no-saccade block. Therefore, the difference in oculomotor activity between the blocked saccade (eye movement) and no-saccade conditions (no movement) should not matter for the final experimental contrast, because it should be subtracted away anyway in calculating the preview effect. Still, to further reduce the contribution of oculomotor activity to the EEG signal we applied ICA and we ran the whole analysis both with and without ICA. As it is common for an event-related potential analysis where temporal precision is important, we avoided a high-pass filter (cf. Acunzo et al., 2012) for the main analysis and conducted the ICA in a separate pipeline with an additional high-pass filter (Hamming windowed sinc FIR, edge of the passband: 1 Hz, −6 dB cutoff frequency: 0.5 Hz). This filter was applied after down-sampling and before low-pass filtering (Dimigen, 2020; Winkler et al., 2011). The precise ICA algorithm was Infomax (Bell & Sejnowski, 1995) with the PCA option activated to account for the reduced rank of some of the datasets that contained interpolated channels and it was run on non-baseline-corrected epoched and clean data which had been visually inspected for major artifacts. The resulting ICA sphere and weight parameters were transferred to the epoched data in the original processing pipeline that lacked the high-pass filter and IC activations were recomputed. Eye-movement-related components were determined based on the variance ratio of component activation during periods of eye movements (blinks and saccades) versus periods of fixations (Plöchl et al., 2012).

The statistical structure for all analyses in this study was a 2 × 2 design with the within-participants factors Preview (valid vs. invalid) and Saccade condition (saccade vs. no-saccade/stable gaze).

## Results

### Manual responses

#### Response times

**Fig. 2:**
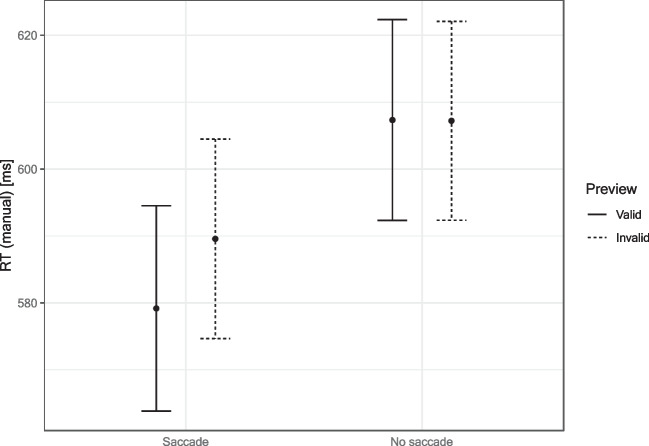
Manual response times in the tilt discrimination task with the target face. The saccade block showed a preview effect with faster responses in valid compared with invalid trials. In the no-saccade block, this effect was gone. For statistics see main text. Error bars denote Morey-factor corrected 95% within-participant CIs

In the saccade condition, manual response times to the target tilt were measured with respect to the fixation onset. In the no-saccade condition, they were measured with respect to the target onset, which can be considered a simulated fixation onset. As expected based on the idea that saccade execution is required for a preview effect, a significant Preview × Saccade interaction, *F*(1, 25) = 7.21, *p* = .013, η_p_^2^ = .22, indicated that manual responses were faster after valid compared with invalid preview only in the saccade condition (valid 579 ms, invalid 590 ms), *t*(25) = 3.02, *p* = .006, *d* = 0.59; see Fig. 2). In the no-saccade condition, manual response times to the target tilt were the same in valid and invalid preview conditions (valid 607 ms, invalid 607 ms). The main effect of the saccade condition was not significant, *F*(1, 25) = 3.50, *p* = .073, η_p_^2^ = .12.

#### Error rates

With the staircase procedure, the percentage of errors across all trials was average at 9%, ranging across participants from 4 to 14%. For the statistical analysis of error rates, we only considered trials that were included in the EEG analysis and the corresponding error trials. Error rates were minimally lower in the valid preview (8.7%) compared with the invalid preview condition (9.2%), *F*(1, 25) = 4.95, *p* = .035, η_p_^2^ = .17. The Preview × Saccade interaction and the saccade main effect were not significant, *F*(1, 25) = 0.54, *p* =.46 9, η_p_^2^ = .02, and *F*(1, 25) = 0.31, *p* = .583, η_p_^2^ = .01, respectively.

### Gaze behavior

The experimental task was gaze-contingent and required the same procedure for each participant. Still, there was room for small deviations in gaze behavior in ways that could be either theoretically important or that could present confounds for any neural effects. The ways in which gaze behavior could differ between conditions was in terms of saccadic latencies and amplitudes in the saccade condition as well as in terms of the duration of the first fixation on the target face in both saccade and no-saccade conditions.

#### Saccade latencies and amplitudes

Per design, saccade latencies could only be measured in the saccade condition. They were on average 7 ms faster in valid (265 ms) compared with invalid trials (272 ms), *t*(25) = −2.12, *p* = .044, *d* = −0.42, and they tended to be by 0.04° (i.e., less than a tenth of a degree of visual angle) larger in valid than in invalid trials, *t*(25) = 2.04, *p* = .053, *d* = 0.40. Because the invalid previews were exclusively inverted faces, these two results mean that saccades to upright faces were a bit faster and possibly larger than saccades to inverted faces. That small difference is perhaps not surprising, as upright faces are potentially more salient and interesting. This pattern would be of concern for interpreting fixation-locked potentials if the effects had not been negligibly small. The difference in the fixation-locked potentials is in the order of magnitude of about 1 µV for saccades of 2.5° amplitude (Dimigen et al., 2011; Kaunitz et al., 2014). The amplitude difference of 0.04°, thus translates to a potential difference of 0.016 µV. Similarly, the difference in saccade latency was 7 ms, which is comparatively short considering that we evaluated the preview effect in the N170 time period for a duration of 85 ms (i.e., 165–250 ms). The differences in saccade latency and amplitude should then be negligible in terms of their effects on the ERPs. If these differences had been larger, they would have presented severe confounds for later ERP effects (Dimigen et al., 2011; Kaunitz et al., 2014; Ries et al., 2018).

#### First fixation duration

For the saccade condition, the duration of the first fixation was clearly defined. For the no-saccade condition, this was less clear because there was by instruction no fixation onset in the whole trial. However, for some participants we observed during the experiment that they moved their eyes after the target had appeared at the screen center. Thus, we defined a first fixation duration in the no-saccade condition as the time period between simulated target onset and the time point at which a first (unintentional) saccade was made in order to see whether participants had eventually maintained stable gaze. This measure showed a strong main effect of the saccade/no-saccade contrast, *F*(1, 25) = 14.46, *p* = .001, η_p_^2^ = .37. First fixation durations were on average 601-ms long in the saccade condition and more than twice as long, 1,238 ms, in the no-saccade condition. No other effects were significant; preview *F*(1, 25) = 2.15, *p* = .155, η_p_^2^ = .08, Preview × Saccade condition interaction, *F*(1, 25) = 0.48, *p* = .493, η_p_^2^ = .02. Because the fixation duration in the no-saccade condition was a rather artificial measure that yielded comparatively large values which might not warrant inferences about actual visual processing, we separately analyzed the fixation durations in the saccade condition. Within the saccade condition, we found shorter first fixation durations in the invalid (536 ms) compared with the valid preview condition (639 ms), *t*(25) = 5.68, *p* < .001, *d* = 1.11.

### Fixation-related and event-related potentials

**Fig. 3:**
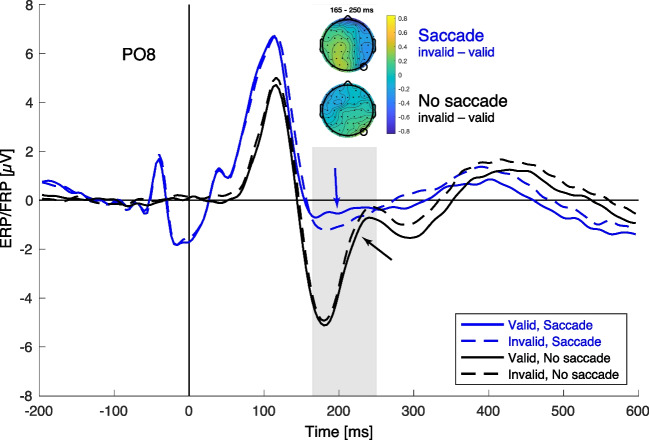
Event-related potentials at electrode PO8 time-locked to the target fixation onset (saccade condition) and the target display onset (no-saccade condition). The average amplitude of the preview effect (invalid minus valid) in the N170 time period was evaluated at 165–250 ms, illustrated by the shaded grey area, and by separate scalp maps for saccade and no-saccade conditions. Arrows point to the decisive differences between invalid (dashed) compared with valid (solid) conditions in the waveforms. (Color figure online)

In the saccade condition, we time-locked the EEG signal to the fixation onset, in the no-saccade condition to the target onset at screen center. The respective fixation-locked and target-locked waveforms are illustrated in Fig. 3 for right hemisphere electrode PO8, at which we had previously found clear preview effects for stimuli in the left visual hemifield (Experiment 2 in Huber-Huber et al., 2019). As can be seen from this figure, the saccade conditions seemed to show a larger P1, and the face-related N170 was almost absent compared with the no-saccade conditions. Importantly, as expected, based on the hypothesis that saccade execution is required for a trans-saccade preview effect, the invalid preview condition showed a more negative deflection than the valid preview condition in that time window when we had previously observed robust preview effects (i.e., 165 to 250 ms after fixation onset; Huber-Huber et al., 2019); but only within the saccade and not within the no-saccade condition. This observation was statistically evaluated by a 2 × 2 repeated-measures analysis of variance (ANOVA) on mean amplitudes across the 165–250-ms time window, which showed the hypothesized significant Preview × Saccade condition interaction, *F*(1, 25) = 7.37, *p* = .012, η_p_^2^ = .23. Follow-up *t* tests, however, revealed a more surprising pattern. Within the saccade condition, the preview effect was statistically not significant, *t*(25) = 1.80, *p* = .084, *d* = 0.35, but within the no-saccade condition, it was significant, *t*(25) = 2.49, *p* = .020, *d* = 0.49, and, as can be seen from Fig. 3, its direction was to the opposite, with a slightly more negative deflection in the valid than in the invalid preview condition. This pattern suggests that the significant Preview × Saccade interaction, which indeed statistically confirms our hypothesis of a larger preview effect in the saccade than in the no-saccade condition, is driven by a slightly reversed preview effect pattern in the no-saccade condition together with a visually well-discernable (Fig. 3) but statistically less robust preview effect in the saccade condition.

The main effect of preview was not significant, *F*(1, 25) = 0.17, *p* = .682, η_p_^2^ = .01. The main effect of the saccade factor was significant, *F*(1, 25) = 15.01, *p* = .001, η_p_^2^ = .38, and confirmed the considerably larger N170 (peak by about 4 µV more negative) in the no-saccade compared with the saccade condition (Fig. 3).

As Fig. 3 revealed, there was still substantial eye-movement-related activity around the time of fixation onset in the saccade condition despite the application of ICA. This is not unexpected, because ICA does not completely remove oculomotor signals and the residual activity becomes particularly apparent in saccade- or fixation-locked data (Dimigen, 2020). To see whether and how the results depended on the application of ICA, we reran the analysis without ICA. This analysis showed the same Preview × Saccade interaction, *F*(1,25) = 7.69, *p* = .010, and preview main effects, *F*(1,25) = 0.21, *p* = .654. Follow-up *t* tests to the interaction were the same, too, with the preview effect in saccade, *t*(25) = 1.21, *p* = . 071, and in no-saccade conditions, *t*(25) = 2.34, *p* = .027, in opposite direction. The only exception was the main effect of the saccade condition which was not significant anymore, *F*(25) = 0.27, *p* = .606, and showed that the N170 was overall more similar in saccade and no-saccade conditions without applying ICA.

## Discussion

In this study, we investigated whether the neural and behavioral preview effects that we have reported previously (Huber-Huber et al., 2019; Huber-Huber & Melcher, 2021) require saccadic eye movements or whether they are not directly associated with saccades and also occur in the absence of active gaze behavior. In line with our hypothesis that active gaze behavior is crucial, we found a larger preview effect in the N170 time period (i.e., a more negative contrast invalid minus valid preview) in the active viewing condition, in which participants made a cued saccade to an extrafoveal face, compared with a no-saccade control condition, where participants maintained fixation throughout each trial and the extrafoveal face reappeared after a simulated saccadic latency (plus saccade duration) at the screen center. This finding demonstrates that active gaze behavior substantially increases the impact of the preview information on postsaccadic processing.

The increased preview effect in the saccade condition is very likely related to the anticipatory processing of information from the saccade target location that comes with saccade execution (cf. Huber-Huber et al., 2021b; Hunt & Cavanagh, 2009; Melcher, 2007, 2011; Sun & Goldberg, 2016). Interestingly, this type of presaccadic enhancement has recently been identified as a form of attention that can be systematically dissociated from covert visual attention (Li et al., 2021). Similar theoretical notions have evolved in ERP research by taking advantage of EEG and eye-tracking coregistration. An ERP component that has classically been associated with covert visual attention, the N2pc (Eimer, 1996; Luck & Hillyard, 1994), has been found to occur not necessarily but only in a very much task-dependent way before eye-movements (Buonocore et al., 2017; Huber-Huber et al., 2016; Talcott & Gaspelin, 2021; Talcott et al., 2023; Weaver et al., 2017), which indicates that covert visual attention is independent of presaccadic attentional enhancements. Our results perfectly tie in with this dissociation. The preview effect was reduced, even reversed, when there was no saccade, and we had previously found the same preview effect in an experimental design with two bilateral instead of one face stimulus (Experiments 1 and 2 in Huber-Huber et al., 2019). If the preview effect resulted from covert visual attention, it should be equally present in an experimental design with a single target face and no-saccade, because covert visual attention should still be attracted to the single target face. Moreover, finding a largely comparable preview effect with one and two bilateral target faces suggests that covert attention did also not enhance the preview effect. Thus, it seems very plausible that the preview effect results from distinctly presaccadic, but not covert, attention.

However, based on the details of the present pattern of results it is difficult to tell whether active gaze behavior does not only increase the neural preview effect in the N170 time window but is strictly speaking also *necessary* for the preview effect in the sense that there would not be any preview effect without active gaze behavior. Follow-up tests to the significant Preview × Viewing condition interaction showed that in the no-saccade condition, there was a small yet significant preview effect in the N170 time period, and this preview effect was in the opposite direction with a more negative deflection in valid than in invalid preview conditions. This finding was rather unexpected, and it might indicate that some sort of very late preview face inversion effect carried over to the postfixation period. The face inversion effect consists in a larger negativity for inverted compared with upright faces (Rossion et al., 1999), which would translate into a larger positivity for invalid than for valid preview trials in our pretarget baseline-corrected data and therefore could counteract any preview effect. However, this explanation goes slightly against other aspects of the results which suggest that face configuration, such as upright/inverted, was not processed much before the target onset in the no-saccade condition (see below). In contrast, in the saccade condition, the preview effect in the N170 time period was statistically not significant but numerically in line with the usual preview effect direction (Buonocore et al., 2020; de Lissa et al., 2019; Huber-Huber et al., 2019). This discrepancy in statistical significances suggests that there was more noise in the saccade than in the no-saccade condition. Moreover, the very small and reversed yet significant preview effect in the no-saccade condition suggests that some sort of extrafoveal-to-foveal information integration takes place also in the absence of active gaze behavior (Contemori et al., 2022; Williams et al., 2008). The mechanisms behind this effect are probably related to the mechanisms that lead to residual trans-saccadic-perception-like effects in the absence of eye movements (Paeye et al., 2018; Valsecchi & Gegenfurtner, 2016). To what extent exactly this type of information integration is similar to or based on the same trans-saccadic integration processes that result in the preview effect, remains to be answered.

The unexpected pattern of a greater yet not significant preview effect in the saccade condition, and a reversed yet significant preview effect in the no-saccade, might also be explained by an overall smaller preview effect in the N170 time period in the present experiment. If the preview effect had been generally larger, we would have likely found a statistically significant preview effect within the saccade condition and in the no-saccade condition the unexpectedly opposite direction of the effect would probably have been cancelled out.

There are two main reasons for why there could have been a smaller preview effect in the present experiment and these reasons are supported by comparing the present to our previous work (Huber-Huber et al., 2019). Our previous work showed a larger preview effect and contained an internal replication which makes the previous findings more trustworthy and makes us believe that the smaller and less robust preview effect in the current study could originate from differences in the experimental design. First, in contrast to our previous work, in the present study, we only had upright target faces. Having only upright targets could make participants believe that there would only be upright faces in the whole experiment. There might not be any expectation of seeing an inverted preview face at any time and, thus, the fixation-locked brain responses to the upright targets might be more uniform and less dependent on the, sometimes inverted, preview faces. Moreover, the preview face was task-irrelevant, since the task was to report a small tilt in the target item which was not present in the preview. In contrast, reading studies find a preview effect with the same experimental design that manipulates the preview-relevant feature only in the preview but not in the target stimulus. However, readings studies differ in yet another way from our study here. We have a limited set of face stimuli which could be learned in the course of the experiment so that the target stimuli further into the experiment are at some point not new anymore. Reading studies usually use hundreds of different words (e.g., Hutzler et al., 2007; Kornrumpf et al., 2016), which means that each particular target stimulus in a reading study is less predictable than the targets in our study. With less predictable targets, participants might put more weight on the preview, which could increase its impact on postsaccadic processing and therefore lead to a larger fixation-locked preview effect. The second reason that supports the finding of a smaller preview effects is that, in contrast to our previous work, here we had a staircase procedure which allowed for a larger tilt angle for the target face (at max. 10.0°) compared with our previous work (1.8°). This could have led to a larger physical discrepancy between preview face and target face which might have made the valid and invalid conditions less different from each other and, thus, could have led to a smaller preview effect in the N170 time window.

Our hypothesis that the preview effect depends on active gaze behavior was not only supported by fixation-locked potentials, but also clearly evident in the participants’ performance in the tilt discrimination task. Participants judged upon fixation/target onset whether the target face was slightly tilted left or right and the responses in this task were faster after valid compared with after invalid previews. Crucially, this behavioral preview effect was completely abolished in the no-saccade condition, which suggests that saccade execution is not only sufficient but even *necessary* for a behavioral preview effect. The cleaner evidence from the behavioral task compared with the EEG effect is not a surprise considering that the manual response to the target is the final outcome of a set of processes that are only partially reflected in the N170 time window and that act in concert in trans-saccadic perception (Huber-Huber et al., 2019).

Besides the preview-effect modulation in the N170 time window, we made another interesting observation. The N170 in both valid and invalid conditions was much more pronounced in the no-saccade condition than in the saccade condition. This finding has to be interpreted more cautiously, because it could be tightly linked to the differences in eyeball rotation between saccade and no-saccade conditions in the post-fixation period compared with the respective presaccade/pretarget baseline. However, it seems still plausible that this finding does not only reflect oculomotor processes but also sensorimotor processes tied to the saccade (Rao et al., 2016; Sun & Goldberg, 2016), because the difference in the N170 between saccade and no-saccade conditions was increased after applying ICA. Probably, in the saccade condition, some sort of perceptual analysis of the face at least up to the stage of facial configuration (Bentin et al., 1996; Eimer, 2000; Itier & Taylor, 2004; Rossion & Jacques, 2011) took place already before the saccade. In the no-saccade condition, this presaccadic perceptual processing was absent and the corresponding perceptual analysis of the facial configuration seemed to start only after the target face appeared foveally. This logic can explain why the N170, an index of analyzing face configuration (Rossion & Jacques, 2011), is very pronounced after target onset in the no-saccade condition, because the lack of a presaccadic perceptual analysis in the no-saccade condition could make facial analysis start only with foveal target onset. This pattern of results is in line with a study by de Lissa et al. (2019), who similarly reported that the N170 is absent upon first fixation on a face if there was a trans-saccadic preview of that face. In addition to the lack of a presaccadic analysis of the face in the no-saccade condition, the N170 might also have been larger because upcoming foveal input was less predictable in the no-saccade condition. Such an effect could be based on the mechanism that enables the visual cortex to differentiate between self-generated and external motion based on nonvisual input from the thalamic pulvinar (Miura & Scanziani, 2022), or more generally on the efference copy of the motor signal for the eye movement (Cavanaugh et al., 2016; Sommer & Wurtz, 2008).

Our findings about the neural and behavioral preview effects with faces match generally well with the preview effect in reading and suggest that the visual system combines visual input across saccades in more or less the same way for both types of visual behavior. However, there is one clear exception. Reading studies have consistently found that the duration of the first fixation on a target word is prolonged due to an invalid preview (Schotter et al., 2012; Vasilev & Angele, 2017). In contrast, we found that the first fixation on the target face was shorter after an invalid preview. Interestingly, this finding replicates previous experiments (Huber-Huber et al., 2019). To see this correspondence with previous experiments, it is important to consider the following: In our previous study, both the preview and the target face could have been inverted or upright and we did not find a significant preview effect on first fixation durations. Whether preview face and target face orientation matched, did not matter for the duration of the first fixation on the target face. However, we found a main effect of the preview face orientation (upright or inverted), which demonstrates that the preview face was more relevant for the duration of the first fixation on the target than the target itself (cf. Schotter, 2018; Schotter & Leinenger, 2016). In the present study, we only had upright targets, which means that the factor preview (valid, invalid) corresponds to the preview face orientation (upright, inverted), and thus the main effect of preview on first fixation durations equals a main effect of preview face orientation. With this in mind, both our previous work and the present experiment provide consistent evidence that the effect of the extrafoveal preview on the duration of the first foveal target-fixation for face images is in the opposite direction to what is usually found in reading research. Our working hypothesis to reconcile these apparently contradicting findings is to consider how easily task-relevant information can be extracted extrafoveally before fixating the target and how much a follow-up saccade reduces uncertainty with respect to task-relevant information. Consequently, we expect that the direction of the preview effect on first fixation durations scales with stimulus size and eccentricity. In reading studies, the preview word is usually presented closer to the fovea (parafoveally) and is smaller with words extended over e.g. 1–2° of visual angle (Schotter & Leinenger, 2016) than in our preview studies with faces (at 8° eccentricity extending across 2.88°). The closer and smaller preview word in reading studies means that fixating on a single position within a target word better provides task-relevant information. For the more eccentric and larger faces in our studies, fixating a single location within the target face provides less task-relevant information (judging the face tilt) and therefore, after an invalid preview, the duration of the first fixation is shorter and a second fixation is made earlier within the same target face in order to gain more information about the target’s tilt. This hypothesis, however, remains to be tested.

Finally, our study provides an example for a very important methodological issue in EEG and eye-tracking coregistration research. We used Infomax ICA in order to remove the contribution of eye-movement activity from the EEG signal. However, as can be seen from Fig. 3, time-locking the signal to eye-movement events reveals nonzero activity around the time of the fixation onset in the saccade conditions that is certainly related to eye movements, also because this type of activity is completely absent in the no-saccade conditions. This finding illustrates the well-known fact that ICA does not completely remove eye-movement-related activity from the EEG signal (Dimigen, 2020). 

The presence of residual eye-movement activity in the saccade conditions does, however, not have any severe implications for the interpretation of our findings because the experiment was designed with the idea that eye-movement characteristics cancel out in the effect of interest. In the saccade viewing conditions, participants made the same eye-movement to the target in both valid and invalid preview trials. In the no-saccade viewing condition, participants did not make any eye-movements at all. Thus, the fact that participants made a saccade in the active viewing conditions but no saccade in the passive viewing conditions cancels out in the final interaction contrast that compares the preview effect under active viewing to passive viewing conditions. Reanalyzing the data without ICA confirmed that our central finding of a greater preview effect in the saccade than in the no-saccade condition did not strongly depend on how much oculomotor activity was present in the EEG signal.

## Conclusion

In sum, we observed that active gaze behavior considerably increases the preview effect in the N170 time window, and saccadic eye movements were necessary for a preview effect in manual response times. This dependency of the preview effect on active gaze behavior is consistent with theories of active vision that postulate that the visual system uses oculomotor information, e.g. corollary discharge (Cavanaugh et al., 2016) to predict before a saccade what will appear, when, and where on the retina. These additional sensorimotor processes clearly affect the visual processing cascade. To what extent the visual processing cascade is eventually affected and how in particular the timing of visual processing stages fits with the temporal structure of active gaze behavior is a matter of future research (Jensen et al., 2021). The present findings, along with a growing number of studies, suggests that the classic experimental setup with fixed gaze that is typically used in EEG studies is not a good model of visual perception, at least when it comes to investigating its active and predictive nature.

## Data Availability

The datasets generated and analyzed during the current study and the analysis code are available from the corresponding author on reasonable request. This study was not preregistered.
